# The Application of a Modified d-ROMs Test for Measurement of Oxidative Stress and Oxidized High-Density Lipoprotein

**DOI:** 10.3390/ijms18020454

**Published:** 2017-02-21

**Authors:** Fumiaki Ito, Tomoyuki Ito, Chinatsu Suzuki, Tomoyo Yahata, Kazuyuki Ikeda, Kenji Hamaoka

**Affiliations:** 1 Institute of Health Sciences, Sunstar Inc., Osaka 569-1195, Japan; 2 Department of Rehabilitation Medicine, Kyoto Prefectural University of Medicine, Kyoto 602-8566, Japan; rinito@par.odn.ne.jp; 3 Department of Pediatric Cardiology and Nephrology, Kyoto Prefectural University of Medicine, Kyoto 602-8566, Japan; suzuki@koto.kpu-m.ac.jp (C.S.); tomoyo@koto.kpu-m.ac.jp (T.Y.); ikeda@koto.kpu-m.ac.jp (K.I.); khamaoka@koto.kpu-m.ac.jp (K.H.)

**Keywords:** reactive oxygen species (ROS), high-density lipoprotein (HDL), diacron-reactive oxygen metabolites (d-ROMs) test, oxidized high-density lipoprotein, oxidative stress, atherosclerosis

## Abstract

Reactive oxygen species (ROS) are involved in the initiation and progression of atherosclerosis. ROS-derived hydroperoxides, as an indicator of ROS production, have been measured by using the diacron reactive oxygen metabolites (d-ROMs) test, which requires iron-containing transferrin in the reaction mixture. In this study we developed a modified d-ROMs test, termed the Fe-ROMs test, where iron ions were exogenously added to the reaction mixture. This modification is expected to exclude the assay variation that comes from different blood iron levels in individuals. In addition, this Fe-ROMs test was helpful for determining the class of plasma lipoproteins that are hydroperoxidized. Low-density lipoprotein/very low-density lipoprotein (LDL/VLDL) and high-density lipoprotein (HDL) were purified by use of an LDL/VLDL purification kit and the dextran sulfate-Mg^2+^ precipitation method, respectively; their hydroperoxide contents were assessed by performing the Fe-ROMs test. The majority of the hydroperoxides were detected only in the HDL fraction, not in the LDL/VLDL. Further detailed analysis of HDLs by size-exclusion high-performance liquid chromatography revealed that the hydroperoxide-containing molecules were small-sized HDLs. Because HDL was shown to be the principal vehicle for the plasma hydroperoxides, this Fe-ROMs test is a beneficial method for the assessment of oxidized-HDL levels. Indeed, Fe-ROMs levels were strongly associated with the levels of oxidized HDL, which were determined by performing the malondialdehyde-modified HDL enzyme immunoassay. In conclusion, the Fe-ROMs test using plasma itself or the HDL fraction after dextran sulfate-Mg^2+^ precipitation is useful to assess the functionality of HDL, because the oxidation of HDL impairs its antiatherogenic capacity.

## 1. Introduction

Oxidative stress can be caused by excess reactive oxygen species (ROS) and reactive nitrogen species. ROS include superoxide (O_2_^•−^), hydrogen peroxide (H_2_O_2_), hydroxyl radical (^•^OH), and singlet oxygen (^1^O_2_). They are generated by the consumption of O_2_ and thought to cause deleterious oxidative damage to virtually all molecules. These reactive species are not necessarily a threat to the body under normal physiological conditions [1,2], but if the body is not able to remove them to a certain degree, oxidative stress stimulates the formation of atherosclerotic plaques and increases the risk of coronary artery disease, type 2 diabetes mellitus, and atherosclerosis [3,4].

Despite the accumulated knowledge concerning the roles of oxidative stress in many dangerous pathophysiological processes, it is still difficult to measure ROS. Electron spin resonance has been recognized as the most powerful technique for the detection of ROS in the form of free radicals. However, ROS are short lived and do not accumulate to sufficiently high levels to be measured.

ROS oxidize various biological macromolecules such as proteins, lipids, and DNA, thus causing structural and functional changes in them. Among these molecules, lipids in membranes and lipoproteins are prominent oxidation targets. Lipid oxidation generates hydroperoxides, which subsequently undergo fragmentation to produce a broad range of reactive intermediates such as the arachidonic acid-derived prostaglandin F2α isomer isoprostanes (IsoPs), malondialdehyde (MDA), 4-hydroxy-2-*trans*-nonenal (HNE), and 4-hydroxy-2-*trans*-hexenal (HHE) [5,6,7,8,9]. Further modification of these aldehydes yields various secondary oxidation products of protein. For example, dihydropyridine (DHP)-type adducts including DHP-lysine (*S*)-2-amino-6-(3,5-diformyl-4-methyl-4*H*-pyridin-1-yl)-hexanoic acid result from the covalent chemical adduction of MDA with protein-bound lysine residues [10,11]. Many methods have been developed for measuring ROS-induced oxidation, based on the determination of oxidation products such as hydroperoxides, IsoPs, and DHP-lysine. Among these products, hydroperoxides are thought to be useful targets to assess oxidative stress, because the hydroxyl radical is the most reactive form of ROS and can initiate lipid peroxidation by attacking polyunsaturated fatty acids (PUFAs) [12]. In addition, hydroperoxides are produced by reactions of peroxynitrate and singlet oxygen with lipids, amino acids, peptides, and proteins [13,14].

The diacron reactive oxygen metabolites (d-ROMs) test can quantify the oxidative stress status by measuring hydroperoxides and has been introduced to analyze their levels in serum or plasma. This test is based on the principle that iron ions released from serum proteins under acidic conditions (pH 4.8) stimulate the conversion of hydroperoxides to alkoxyl and peroxyl radicals, which subsequently react with the chromogen diethyl *p*-phenylene diamine hydrochloride. This test is a simple and an easy way of detecting hydroperoxides, but there are some factors limiting its widespread use: (1) a high-priced machine (automatic biochemical analyzer) or specifically designed machine (FRAS4, Wismerll Co., Tokyo, Japan) is required; (2) the chemical entities measured are still elusive; and (3) the antioxidant substances and blood levels of metal ions, such as iron, may affect the measurement accuracy. Therefore, simpler and more reliable methods for analysis of hydroperoxides are required.

When blood samples are used for the determination of hydroperoxides, lipoproteins are the most likely entities measured by the d-ROMs test. Oxidatively modified low-density lipoprotein (oxLDL) is one form of modified LDL that has been demonstrated to be present in vivo [15], specifically in atherosclerotic lesions [16], and has been implicated in the pathogenesis of atherosclerotic diseases [17,18]. On the other hand, another subclass of lipoproteins, high-density lipoprotein (HDL), is known to be atheroprotective due to its abilities to reversely transport cholesterol and remove oxidized lipids from oxLDL [19,20,21]. However, these abilities are decreased or modulated when HDL is oxidized [22,23,24,25]. Therefore, having methods available to analyze the oxidative stress status of HDL as well as that of LDL is valuable in daily clinical practice.

In this study, we developed a modified d-ROMs test that could serve as a useful basis for evaluating the oxidative status of plasma. Furthermore, we studied whether our d-ROMs test would be applicable for the assessment of oxLDL and oxidized-HDL (oxHDL) levels of human plasma. This work proves the applicability of our modified test for evaluating the level of oxHDL.

## 2. Results

### 2.1. The Measurement of Hydroperoxides in Plasma by the Diacron-Reactive Oxygen Metabolites (d-ROMs) and Fe-ROMs Tests

**Figure 1:**
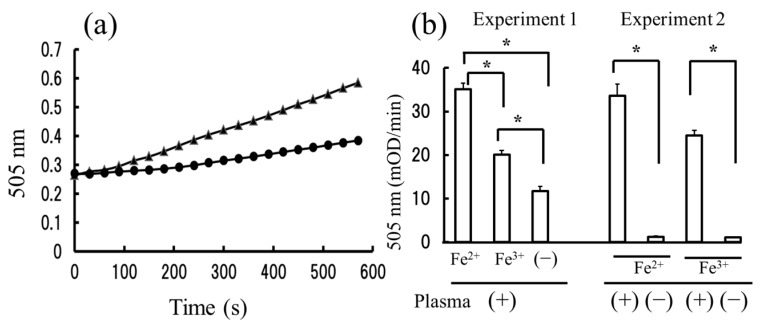
The effect of iron ions on the measurement of reactive oxygen metabolites. (**a**) Time courses of absorbance changes at 505 nm after the addition of plasma from a healthy subject and *N*,*N*′-diethyl-*p*-phenylenediamine were determined in the presence (▲) and absence (●) of 100 µM Fe^2+^ as described in the (Section 4); (**b**) Increased rate of absorbance (mOD/min) during 120 to 300 s was determined after the addition of *N*,*N*′-diethyl-*p*-phenylenediamine, and iron ions (Fe^2+^ or Fe^3+^) in the presence of plasma (Experiment 1) or in the presence or absence of plasma (Experiment 2). The results are expressed as the mean ± standard deviation (*n* = 5). A symbol (*) represents a statistically significant difference (*p* < 0.001). When the plasma samples from two other healthy subjects were used, similar effects of Fe ions were observed.

The conversion of hydroperoxides to alkoxyl and peroxyl radicals is dependent on metal ions such as Fe and Cu. In the d-ROMs test, these ions are supplied by metal-containing proteins in blood samples [26,27]. Instead, here we added iron ions exogenously to the reaction mixture, expecting that the levels of hydroperoxide could be measured independent of the concentrations of endogenous iron in the subjects. This modified method was named the “Fe-ROMs test” after the addition of Fe ions. In total, 200 µL of 0.1 M acetate buffer (pH 4.8) containing 100 mM *N*,*N*′-diethyl-*p*-phenylenediamine and an aliquot of plasma were added into each of the 96 wells in a micro-plate and incubated in the presence or absence of 100 µM Fe^2+^ for 10 min at 37 °C. As shown in Figure 1a, the absorbance at 505 nm linearly increased after a lag time of 60 to 120 s irrespective of the presence or absence of Fe^2+^, and its rate of increase during the 120 to 300 s was 37.005 mOD (optical density ×10^−3^)/min in the presence of Fe^2+^ and 11.868 mOD/min in the absence of it. This Fe effect was also observed in the presence of Fe^3+^ (Figure 1b, Experiment 1), and the rate of increase was nearly zero if the plasma was omitted from the reaction mixture (Figure 1b, Experiment 2).

### 2.2. The Strong Correlation between Levels of Hydroperoxides Measured by the d-ROMs and Fe-ROMs Tests

**Figure 2:**
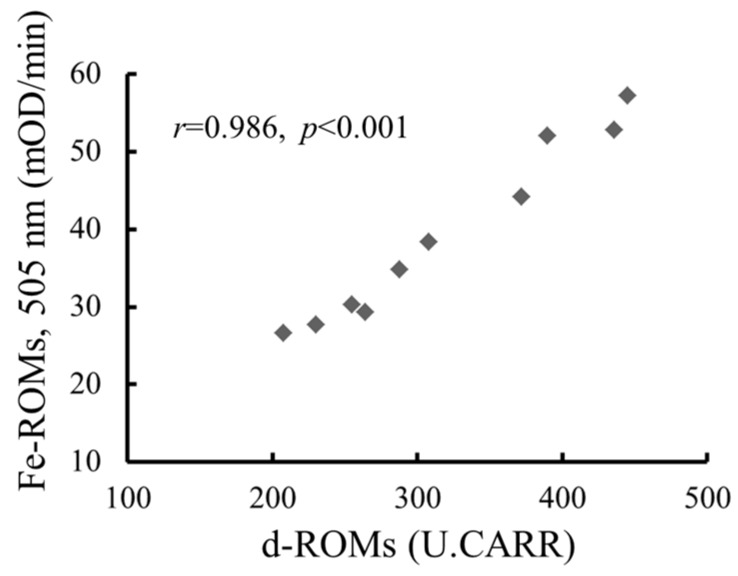
The correlation of Fe-ROMs values with diacron reactive oxygen metabolites (d-ROMs) values. The oxidative stress was evaluated by measuring plasma samples from 10 male patients. The rates of increase in absorbance at 505 nm were determined in the presence (Fe-ROMs test) and absence (d-ROMs test) of Fe^2+^, as described in the Section 4. The correlation between the values obtained from the d-ROMs and Fe-ROMs tests was expressed by Pearson’s correlation coefficient (*r* = 0.986, *p* < 0.001). U.CARR, Carratelli units.

We next measured the levels of hydroperoxide in the plasma from 10 subjects with experience of acute febrile disease by using the original d-ROMs test and our modified one (the Fe-ROMs test). For this experiment, we chose subjects with acute febrile disease because they show a wide range of d-ROMs levels from normal to high. Indeed, their hydroperoxides levels ranged from 205 to 430 Carratelli units (U.CARR) (Figure 2). The levels obtained by the original and the Fe-ROMS tests showed a strong correlation (*r* = 0.986), although the ratios of Fe-ROMs levels to d-ROMs levels tended to become higher as the d-ROMs levels increased. The correlation between both levels was also strong in five healthy subjects (*r* = 0.896, Figure S1).

### 2.3. The Fe-ROMs Test Detects the Oxidation of Plasma Molecules of High Molecular Weight

The Fe-ROMs test has some advantages over the original one. In the original test, both hydroperoxides and iron ion are supplied by plasma; but in the Fe-ROMs test, iron ions are supplied exogenously in the reaction mixture. Therefore, the Fe-ROMs test allowed us to fractionate hydroperoxide-containing molecules from plasma without paying attention to the presence of iron-supplying molecules. We first separated plasma into filtrate and residual fluid by using an Amiconultra-0.5 (100 kDa). The recovery of hydroperoxides in the residual fluid, which was determined by the Fe-ROMs test, was 99.3% (the average recovery of three independent experiments), indicating that most of the hydroperoxidized molecules were included among the macromolecular components.

### 2.4. Defining the Macromolecular Components Oxidized in Plasma: Oxidized Macromolecular Components Are Not Low-Density Lipoprotein/Very Low-Density Lipoprotein (LDL/VLDL)

Oxidative stress targets a variety of molecules in plasma. Among them, lipids, especially PUFAs, are most subjected to oxidative stress, leading to lipid peroxidation. Lipoproteins are macromolecular complexes of phospholipids, free cholesterol, cholesterol esters, triglycerides, and apolipoproteins. Because phospholipids, cholesterol esters, and triglycerides contain PUFAs, it is most likely that hydroperoxides are present in plasma lipoproteins. We then isolated the low-density lipoprotein/very low-density lipoprotein (LDL/VLDL) fraction from the plasma by using an LDL/VLDL Purification Kit. A dextran solution was first added to the plasma to selectively precipitate LDL/VLDL from the plasma, and the pellet obtained by centrifugation was used for the subsequent LDL/VLDL purification. We detected less than 3% of hydroperoxides in the purified LDL/VLDL as compared to the amount in the original plasma sample, indicating that the hydroperoxide level in LDL/VLDL was too low to be detected by the Fe-ROMs test. In contrast, all the activity (105%) was detected in the supernatant obtained after centrifugation following the addition of the Dextran solution, indicating that HDL was probably the major carrier of hydroperoxides in plasma.

### 2.5. The Oxidation of HDL Is Detected by the Fe-ROMs Test

**Figure 3:**
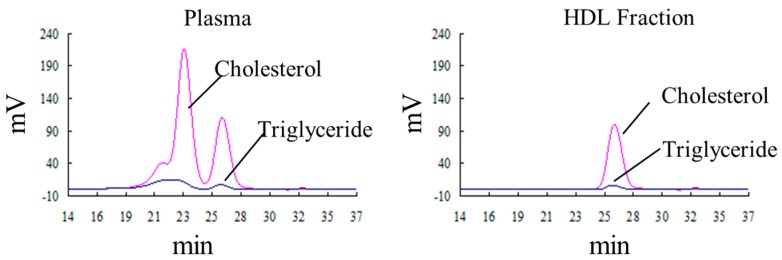
The lipoprotein analyses of plasma and high-density lipoprotein (HDL) fraction by gel-permeation high-performance liquid chromatography (GP-HPLC). The HDL fraction was isolated from the plasma of a healthy subject by using the dextran sulfate/Mg^2+^ method as described in the Section 4. The plasma and isolated HDL fractions were analyzed by using a dual detection GP-HPLC system for lipoprotein analysis as described in the Section 4. Cholesterol and triglycerides concentrations were monitored for lipoprotein profiling. The retention times of the low-density lipoprotein (LDL) and HDL standard materials were 23.2 and 26.0 min, respectively.

We then isolated the HDL from the other lipoproteins with a combination of dextran sulfate and Mg^2+^, and assayed its activity by performing the Fe-ROMs test. All the activity (104%) was recovered into the supernatant after the dextran sulfate-Mg^2+^ precipitation procedure, suggesting that the hydroperoxide-containing molecule was HDL. The lipoprotein separation was quite sensitive to the molecular size of the dextran sulfate, the concentrations of dextran sulfates and Mg^2+^, and the temperature. Consequently, we evaluated the lipoprotein profiles of the plasma and the supernatant after performing the dextran sulfate-Mg^2+^ precipitation by using gel-permeation high-performance liquid chromatography (GP-HPLC). As shown in Figure 3 and Table 1, the lipoprotein profiles revealed that the plasma contained four major classes of lipoproteins including VLDL, LDL, and HDL, but the supernatant contained only HDL. More detailed analysis of the lipoproteins (Table 1), in which each of these four major classes were divided into sub-classes on the basis of the lipoprotein particle size, shows that all sizes of HDL were recovered in the supernatant. Furthermore, the supernatant contained only small amounts of other lipoproteins including small LDL and very small LDL. This is an important finding, because a small dense LDL, which is associated with an increased risk of atherosclerotic disease, has been reported to be vulnerable to oxidation [28,29,30].

### 2.6. The Fe-ROMs Test Is a Useful Method for the Determination of Oxidized High-Density Lipoprotein (HDL)

**Figure 4:**
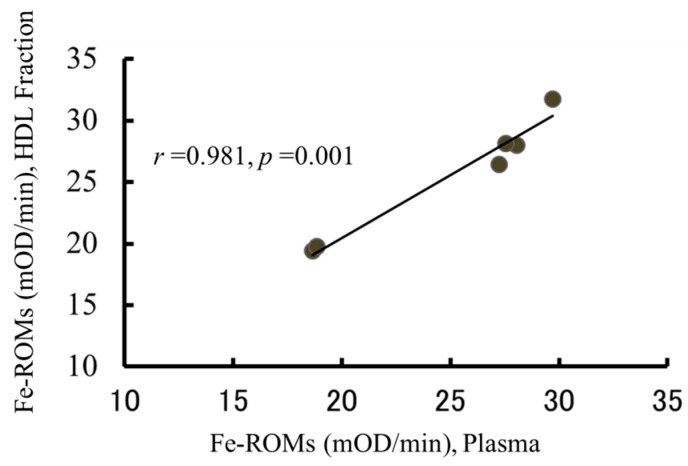
The correlation between the Fe-ROMs levels in the plasma and HDL fraction. HDL fractions were isolated from the plasma of six healthy subjects (aged 27–69 years), as described in Figure 3. The Fe-ROMs values were then measured by using these plasma samples and isolated HDL fractions. The correlation between the Fe-ROMs levels in the plasma and isolated HDL fractions is expressed by Pearson's correlation coefficient (*r* = 0.981, *p* = 0.001).

As described above, almost all the plasma hydroperoxides were detected in the HDL fraction. Because the levels of Fe-ROMs in the plasma were different from subject to subject, it was interesting for us to compare Fe-ROMs levels in the plasma and its dextran sulfate-supernatant in many subjects. Figure 4 shows that the correlation between Fe-ROMs levels in both samples from six healthy men was very strong. Further, a significant correlation was maintained between both samples from 10 subjects with acute febrile disease (*r* = 0.924, Figure S2), indicating that the hydroperoxidized HDL in many subjects can be estimated from the plasma’s Fe-ROMs levels.

### 2.7. Small-Sized HDL Is a Major Oxidized Molecule in Plasma

**Figure 5:**
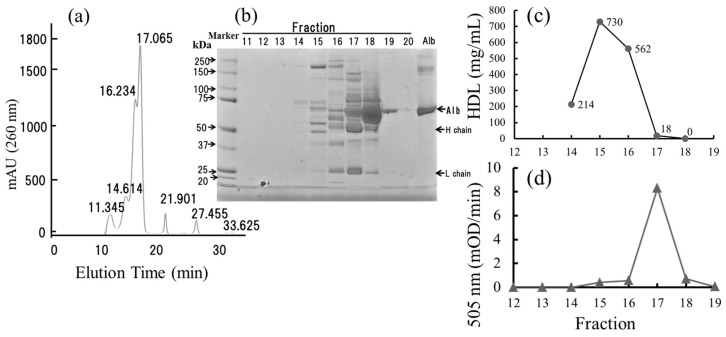
The HPLC analysis of the carrier of hydroperoxides. (**a**) 50 microliters of plasma from a healthy subject was separated on an OH pak SB-804HQ column at a flow rate of 0.5 mL/min and fractionated at 1 min intervals. The numbers on each of the peaks indicate the elution time (min); (**b**) The eluted fractions were collected in 0.5 mL fractions and analyzed by 4%–12% sodium dodecyl sulfate poly-acrylamide gel electrophoresis (SDS PAGE) as described in the Section 4. Each fraction was 0.5 mL in volume. Fraction 11 was collected from elution time 10 to 11 min, fraction 12 from 11 to 12 min, and additional fractions in the same way. Albumin is a major serum protein and was eluted mainly in fraction 18 (elution time is 17–18 min). Alb, Albumin; H chain, Immunoglobulin heavy chain; L chain, Immunoglobulin light chain; (**c**) Each eluted fraction (fractions 14–18) was used for the measurement of the HDL concentration (●), as described in the Section 4. The HDL concentrations were expressed in numbers (214, 730, 562, 18, and 0) on each figure; (**d**) Each eluted fraction (fractions 12–19) was used for the measurement of the Fe-ROMs values.

**Figure 6:**
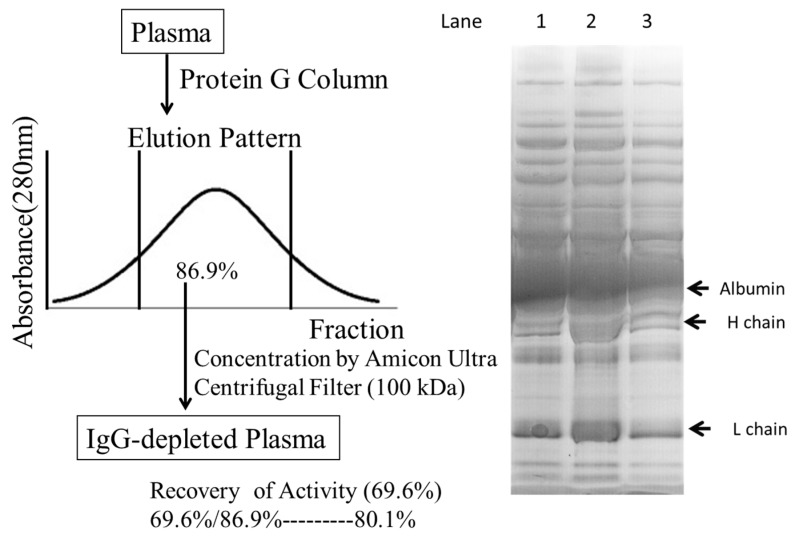
The preparation of IgG-depleted plasma and its Fe-ROMs value. **Left** panel: Plasma from a healthy subject was loaded onto a Protein G Sepharose 4 Fast Flow column, as described in the Section 4. The eluate was assayed for the determination of absorbance at 280 nm, and 86.9% of it was concentrated to obtain IgG-depleted plasma. Recovery of the Fe-ROMs value in IgG-depleted plasma was 69.6% as compared with the value for plasma; **Right** panel: Plasma and IgG-depleted plasma were analyzed on the 4%–12% SDS/PAGE as described in Figure 5. Lane 1: IgG-depleted plasma (44 µg protein), Lane 2: Plasma (43 µg protein), and Lane 3: IgG-depleted plasma (22 µg protein).

We next fractionated the plasma from a healthy subject by using HPLC and defined the molecules that had been hydroperoxidized. As shown in Figure 5a,b, the highest peak (eluted at 17.065 min and collected into Fraction 18) and the second highest one (16.234 min and Fraction 17) were identified as albumin and IgG, respectively, by sodium dodecyl sulfate poly-acrylamide gel electrophoresis (SDS-PAGE) analysis. On the other hand, HDLs were eluted in Fractions 14–17 (Figure 5c). However, hydroperoxide-containing molecules were not eluted coincidentally with the majority of HDLs and were detected only in Fraction 17 (Figure 5d). Because not only small HDL but also IgG were included in Fraction 17, we examined the involvement of IgG in the activity. As summarized in Figure 6, when IgG was removed from the plasma by protein G column chromatography, the recovery of the Fe-ROMs level in IgG-depleted plasma was 80.1%. Because we often experienced a loss of activity during the concentration procedure using Amicon Ultra centrifugation, we concluded that IgG had no critical effect on the Fe-ROMs levels. Therefore, we considered HDL, in particular the small HDL, most likely to be the major carrier of hydroperoxides in plasma.

### 2.8. The Oxidation of HDL Can Be Measured by the Malondialdehyde (MDA)-HDL Enzyme-Linked Immunosorbent Assay and the Fe-ROMs Test

**Figure 7:**
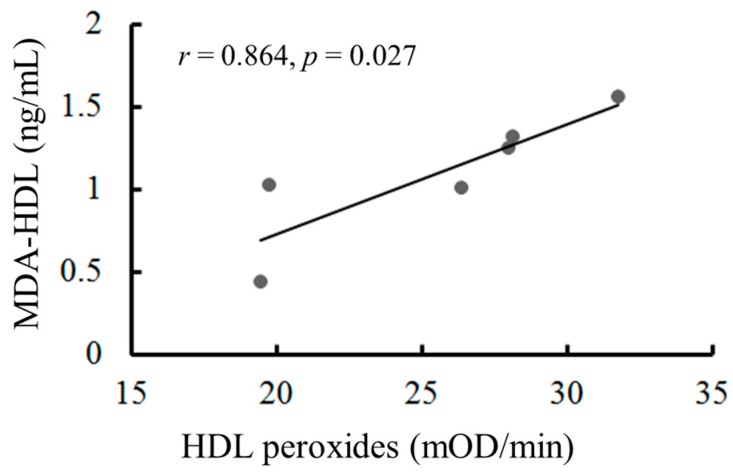
The measurement of oxidized HDL levels by the Fe-ROMs test and the enzyme-linked immunosorbent assay (ELISA). The HDL fractions were isolated from the plasma of six healthy subjects (aged 27–69 years) by using the dextran sulfate-Mg^2+^ method as described in the Section 4. Each of the isolated HDL fractions was used for the measurement of HDL peroxides by use of the Fe-ROMs test and for the measurement of MDA-HDL by use of the ELISA. The correlation between the levels of oxidized HDL, which were measured by the Fe-ROMs test and the ELISA, is expressed by Pearson's correlation coefficient(*r* = 0.864, *p* = 0.027).

The oxidation of HDL causes a reduction in the ability of HDL to promote cholesterol efflux [21,22,23]. Therefore, monitoring circulatory levels of oxHDL is critical to the diagnosis of atherosclerosis. Oxidative stress generates hydroperoxides in HDL and subsequently forms reactive aldehyde products, such as MDA. MDA attaches covalently to the amino groups of lysine residues of HDL protein components (MDA-HDL). Therefore, an enzyme-linked immunosorbent assay (ELISA) for MDA-HDL has been developed for the determination of oxHDL. We obtained HDL fractions from six healthy male subjects by using the dextran-sulfate-Mg^2+^ precipitation procedure and determined their oxidation levels by performing an MDA-HDL enzyme immunoassay and Fe-ROMs test (Figure 7). Both levels were strongly correlated (*r* = 0.864), although two subjects with similar Fe-ROMs levels (about 20 mOD/min) showed different MDA-HDL levels.

### 2.9. Plasma HDL Concentrations in Subjects with Different HDL Oxidation Levels

As shown in Figure 7, the oxHDL levels differed among the six subjects examined. Because the difference may have occurred due to different plasma HDL concentrations in them, we next determined the concentrations of HDL in four subjects, two of which had higher levels of oxHDL, and the others had lower ones. The lipoprotein profiles (Table 2) and more detailed analysis of HDL (Table 3) indicated no significant difference in the HDL concentrations between four subjects. Thus, the subjects with higher Fe-ROMs values had more hydroperoxidized lipids per HDL molecule.

## 3. Discussion

Because there is a close association between oxidative stress and vascular diseases, we need reliable biomarkers to assess such stress. In the present study we assessed oxidative stress in human plasma by use of a modified d-ROMs test (i.e., the Fe-ROMs test) where iron ions were exogenously added to the reaction mixture. Our Fe-ROMs test had some advantages over the original d-ROMs test: (1) The Fe-ROMs test required a smaller volume of plasma to measure oxidative stress; (2) it gave us an opportunity to define hydroperoxidized target molecules in the blood; and (3) it could exclude the assay variation that comes from different blood iron levels in individuals.

The d-ROMs test is a simple assay marketed for analyzing the total amount of hydroperoxides in serum via Fenton’s reaction. However, earlier reports have indicated that the signal detected in this assay is affected by blood components such as iron ions [31]. The serum iron reference range is about 55–160 µg/dL in men and 40–155 µg/dL in women. Therefore, if we use plasma from people with normal serum iron values (40–160 µg/dL) for the determination of d-ROMs levels, iron in the reaction mixture is calculated at a concentration from 0.14 to 0.57 µM. We have observed that d-ROMs levels are increased by iron ions in a dose-dependent manner up to 100 µM (Figure S1). Thus, d-ROMs levels depend on the levels of hydroperoxides and iron ions in plasma. We anticipated that Fe-ROMs levels are not necessarily correlated with those of d-ROMs. However, the correlation between the levels obtained by the d-ROMs and Fe-ROMS tests was strong in subjects with acute febrile disease, although it tended to be non-linear. This non-linearity may indicate that exogenous iron ions are required for the measurement of blood samples containing high levels of hydroperoxides. In the d-ROMs test, iron ions are supplied mostly from Fe^3+^-binding transferrin protein in blood samples. The affinity of transferrin for ferric iron (Fe^3+^) is extremely high under the extracellular environment (approximately pH 7.4), and weakened in the acidic conditions, leading to Fe^3+^ dissociation from the protein [32]. It thus appears that Fe^3+^rather than Fe^2+^ is mainly used in the acidic conditions of the d-ROMs test. Fe^3+^ increased absorbance at 505 nm dose-dependently, but to a lesser extent than Fe^2+^. Therefore, the d-ROMs test might not be strongly influenced by different blood iron levels in individuals. However, we recommend adding exogenous iron ions to the reaction mixture, because adding even a small amount of Fe^3+^ (i.e., 1 µM) to the reaction mixture changed the absorbance at 505 nm (Figure S3).

There are some criticisms about the reliability of the d-ROMs test as a method for estimating oxidative stress in a serum or plasma, as the concentration of some ions (i.e., Fe, Cu, etc.) in blood samples might interact in the assay reaction and influence the results [31]. Recent studies have excluded the critical interference of these ions on the d-ROMs test [33,34]; the correlation between the concentration of iron ions and the d-ROMs readings was non-significant in 502 bovine samples. However, in the Fe-ROMs test, not only iron ion in blood samples but also exogenous iron ion was present in the reaction mixture. Hydroperoxides in the reaction mixture undergo iron-mediated one-electron reduction and form free radicals, which react with *N*,*N*′-diethyl-*p*-phenylenediamine to form a colored radical that is detectable at 505 nm. Concomitantly, the formation of free radicals can initiate a radical chain reaction with the hydrocarbon part of an unsaturated lipid molecule. In this study we have observed a continuous increase in absorbance at 505 nm at least until 10 min irrespective of the presence or absence of iron ions (Figure 1). This observation may indicate that the absorbances in the d-ROMs and Fe-ROMs tests were increased in a similar fashion via a radical chain reaction, although their increased rates were very different from each other.

ROS oxidize not only lipids but also other biological molecules such as proteins and DNA. However, lipids, especially PUFAs, are the ones most targeted by ROS [35]. Lipid oxidation products including hydroperoxides of phosphatidylcholine and cholesteryl ester have been detected and characterized in oxLDL and oxHDL [25]. Therefore, lipoproteins are the most likely targets of the lipid oxidation process in plasma. Indeed, when plasma was separated into two fractions, depending on the molecular size by membrane filtration (with a molecular weight cut-off at 100 kDa), almost all of the hydroperoxide-containing molecules were recovered in the high-molecular weight fraction.

The imbalance between circulating levels of lipoproteins (LDL and VLDL) relative to those of HDL is associated with atherogenesis. LDL is a major substrate for oxidation at the arterial wall, and the oxidatively modified form of LDL (oxLDL) is more important than native LDL in the process of atherogenesis [17,18,36,37]. However, oxHDL, but not oxLDL, is detected in plasma by the Fe-ROMs test. This observed uneven distribution of lipid hydroperoxides in plasma lipoproteins may be attributed to the following two possibilities: Firstly, a substantial amount of oxLDL is formed in the blood, but most of its oxidation products are rapidly metabolized into other molecules or removed by HDL. HDL, especially small, dense HDL3, has been reported to potently protect LDL against oxidative stress, and such potent antioxidative activity is related to the transfer of oxidized lipids from LDL to HDL [23,38]. Thus, HDL might function as a sink for oxidized lipids in the blood [39]. As a second possibility, HDL might be more susceptible to oxidation in the blood than LDL. Interestingly, lipid hydroperoxides were reported to accumulate more rapidly in HDL than in LDL in vivo, because of the action of LDL-associated antioxidants such as ubiquinol-10 [40]. As a consequence, LDL lipids are relatively peroxide-free.

Our HPLC analysis revealed that carriers of hydroperoxides were eluted at a position just after the majority of HDLs were eluted and coincidentally with very small HDL (Figure 5d). HDLs are a class of structurally and functionally heterogeneous particles. They are classified on the basis of their density and size, resulting in the large buoyant HDL2 and the small dense HDL3. HDL3c represents a minor sub-fraction of HDL3, accounting for about 6% of the total HDL mass, and its diameter is about 6.7 nm [35,41,42]. It was reported that HDLs, in particular the small dense HDL3, exert anti-inflammatory and antioxidant activities, suggesting that this HDL plays a role as a ROS scavenger in inflammatory sites [37]. Copper(II)-induced oxidation of LDL in the presence of total HDL, HDL2, or HDL3 demonstrated a less well-marked increase of hydroperoxides within LDL, but with a rapid production of hydroperoxides in HDL [43]. Further, HDL3 appeared to be more effective in preventing the hydroperoxidation of LDL. Taken together, our data indicate that it is likely that very small-sized HDL, for example, HDL3c, was strongly hydroperoxidized in the blood and thus detected by the Fe-ROMs test. However, a possibility still exists that HDL sub-fractions other than very small HDL are also hydroperoxidized in plasma, because the recovery of hydroperoxides after HPLC analysis was less than 20%.

HDL retards atherosclerosis via multiple mechanisms, including reverse cholesterol transport (RCT) [44,45], anti-inflammatory effects [46], and antioxidant ability [46]. However, in subjects with atherosclerosis, HDL becomes dysfunctional, which diminishes these anti-atherosclerotic functions. One of the main reasons for this loss of function is oxidative changes in HDL proteins and lipids. Various reports have described that the site-specific oxidation of apoA-1, the major HDL protein, by myeloperoxidase impairs RCT by the ATP-binding cassette transporter A1 pathway [47,48]. Another potential mechanism for generating dysfunctional HDL involves covalent modification of apoA-I by reactive carbonyls such as MDA and acrolein, which can be produced in the body through lipid peroxidation [49]. The antioxidant action of HDL is attributed to HDL-associated lipolytic enzymes such as paroxonase 1, as well as apoA-I [45,50]. Moreover, HDL particles protect against oxidative stress through their capacity to accept phospholipid hydroperoxides from LDL, and to reduce them to inactive hydroxides via the oxidation of methionine residues in apoA-I [23], indicating that the elevated peroxidation of lipids probably contributes to the antioxidant action. Collectively, these observations provide evidence that peroxidation of lipids in HDL is a potential mechanism for generating dysfunctional HDL.

This study showed that HDL was the principal carrier of lipid hydroperoxides in human plasma when the Fe-ROMs test was used for the determination of oxidative stress. This is an important finding because oxHDL, which is a risk factor for developing atherosclerosis, can be assayed by the Fe-ROMs test. Enzyme immunoassay kits have been developed for the detection and quantification of oxHDL in plasma or serum. In these assays, antibodies against the secondary oxidation products of HDL, such as MDA-HDL, have been used. However, these kits are not used routinely in a clinical examination setting because they require much labor and are expensive. On the other hand, our Fe-ROMs test is inexpensive and convenient for the determination of oxHDL. Our study indicated that hydroperoxidized HDL can be estimated from Fe-ROMs levels in plasma. However, as demonstrated by Bowry et al. [51], LDL carries lipid hydroperoxides, although their levels are far lower than those of HDL. Additionally, lipoproteins other than HDL may carry a substantial amount of hydroperoxides in subjects with cardiovascular disease or low antioxidant intake and in heavy smokers. Therefore, we recommend using plasma samples in a primary assay for the determination of oxHDL, and if necessary, supernatants after dextran sulfate-Mg^2+^ precipitation in a secondary assay.

## 4. Materials and Methods

### 4.1. Materials

Fe(NH_4_)_2_(SO_4_)_2_, FeCl_3_, *N*,*N*′-diethyl-*p*-phenylenediamine, dextran sulfate sodium salt (molecular weight range, 36,000–50,000), MgCl_2_, and human serum albumin were purchased from Wako (Tokyo, Japan). Tert-butyl hydroperoxide (70% in water) was purchased from TCI (Tokyo, Japan). NaCl was from Sigma-Aldrich (Tokyo, Japan). HDL Human ELISA (enzyme-linked immunosorbent assay) Kit (ab125961) came from Abcam (Cambridge, UK). A Bolt LDS sample Buffer, Bolt Reducing Agent, and Bolt MOPS SDS Running Buffer were purchased from the Life Technologies Corporation (Carlsbad, CA, USA). The Precision Plus Protein Dual Color Standards were from Bio-Rad Laboratories, Inc. (Hercules, CA, USA). The OH pak SB-804HQ column was obtained from Showa Denko KK (Tokyo, Japan).

### 4.2. Subjects

Eleven healthy male subjects (aged 30.2 ± 14.3) and 10 male patients (age: 15.7 ± 12.1) who had experienced acute febrile disease were briefed about the study protocol and possible risks, signed the informed consent, and voluntarily participated in the present study. The study protocol was approved by the Ethics Committee of Kyoto Prefectural University of Medicine (RBMR-C-983, 9 November 2011). The patients were included in this study, because they showed a wide range of d-ROMs levels from normal to high. Blood samples were collected from the antecubital vein into sodium heparin-containing blood collection tube for the measurement of reactive oxygen metabolites. The blood samples were centrifuged at 4 °C immediately after collection, and the plasma was separated and stored at −80 °C until analysis could be performed.

### 4.3. The Measurement of Reactive Oxygen Metabolites by the d-ROMs and Fe-ROMs Tests

The amount of hydroperoxides present in a 20 µL volume of plasma was estimated by performing the d-ROMs test using the FRAS4 system according to the manufacturer’s instructions (Wismerll Co., Tokyo, Japan). The results obtained from the d-ROMs test were expressed in arbitrary units called “Carratelli units” (U.CARR). In the Fe-ROMs test, 186 µL of 0.1 M acetate buffer (pH 4.8) containing 100 µM Fe(NH_4_)_2_(SO_4_)_2_ or FeCl_3_ was added into each well of a 96-well plate and warmed to 37 °C. Subsequently, 10 µL of 100 mM *N*,*N*′-diethyl-*p*-phenylenediamine and an aliquot (4 µL) of plasma were added into each well of the 96-well plate and reacted at 37 °C. The reaction product was measured by using an xMark microplate spectrophotometer (Bio-Rad Laboratories, Inc.) with the wavelength, reading mode, and kinetic parameter set at 505 nm, kinetic, and rate, respectively. The results of the Fe-ROMs test were expressed as the rate of increase in OD (mOD/min) during a 3 min period from 2 to 5 min after the addition of the plasma, where 1 mOD/min corresponded to 23.7 µM tert-butyl hydroperoxide. Measurements were run in duplicate and a mean value was used in the analyses (intra-assay coefficient of variability = 3.84%).

### 4.4. Fractionation of HDL by the Dextran Sulfate-Mg^2+^ Precipitation Method

The plasma was ultra-filtered by using a Millex-HV syringe filter unit (0.45 µm, PVDF membrane: polyvinylidene fluoride membrane). Part of the filtrate was stored at −80 °C as the plasma fraction, and the rest of it was processed to isolate the HDL fraction as reported previously [52]: 0.9 mL of the filtrate was mixed at room temperature with 0.1 ml of a solution (pH 7.3) containing 1% dextran sulfate sodium salt and 0.5 M MgCl_2_. The mixture was then stood at room temperature for 10 min and centrifuged (1500× *g*) for 30 min at 4 °C. The supernatant was ultra-filtered by passing through a 0.45 µm-Millex-HV syringe filter unit, and the filtrate was then stored as the HDL fraction at −80 °C. The stored plasma and HDL fractions were used for the lipoprotein analysis by using a dual detection HPLC system at Skylight Biotech (Akita, Japan) according to the procedure described by Usui et al. [53].

### 4.5. Lipoprotein Analysis of the Plasma by HPLC

Any turbidity in the plasma samples was cleared by ultrafiltration through a 0.45-µm Millex-HV syringe filter unit. The cleared plasma sample (0.5 mL) was then separated on an OH pak SB-804HQ column (300 × 8.0 mm) with 0.15 mol/L NaCl at a flow rate of 0.5 mL/min. The column temperature was set at 20 °C, and elution was monitored by absorbance at 260 nm. The column eluate was collected in 0.5 mL fractions and used for the Fe-ROMs test, HDL ELISA assay, and SDS-PAGE analysis.

The HDL ELISA assay was done by using an HDL Human ELISA Kit. Briefly, the column eluate (fractions 14–18) was diluted 1:1 with 1× Diluent M; and an aliquot (25 µL) of the diluted sample was added to the wells of a 96-well plate that had been pre-coated with an antibody specific for HDL. After this addition, the assay was done as indicated in the manufacturer’s instructions; and HDL concentrations were calculated from the standard curve.

### 4.6. Protein Analysis by SDS-PAGE

An aliquot (5 µL) of the column eluate was heated at 70 °C for 10 min in a total 40 µL of a Bolt LDS sample Buffer containing Bolt Reducing Agent. It was then applied on a Bolt 4%–12% Bis-Tris Plus gel, and electrophoresed in Bolt MOPS SDS Running Buffer at 200 volts for 35 min. In parallel, 2 µL of the Bio-Rad Precision Plus Protein Dual Color Standards and 5 µL of 1 mg/mL human serum albumin were run. The gels were then washed with ultrapure water, stained for 1 h with Imperial Protein Stain (Thermo Fisher Scientific, Waltham, MA, USA), and destained with ultrapure water. The stained protein bands were detected by using an Image Quant LAS 4000 mini (GE Healthcare Life Sciences, Marlborough, MA, USA).

### 4.7. Protein G Affinity Chromatography

The plasma was cleared by ultra-filtration using a Millex-GV syringe filter unit (0.22 µm, PVDF). The cleared plasma (0.3 mL) was loaded onto a Protein G Sepharose 4 Fast Flow column (gel volume of 2 mL) and eluted with 10 mmol/L phosphate-buffered saline (pH 7.4) at 0.2 mL/min. The eluate was collected in a total of 18 fractions, each containing 0.2 mL. Seven fractions with high absorbance at 280 nm were collected and concentrated by using an Amicon Ultra-0.5 mL 100K (Merck KGaA, Darmstadt, Germany) according to the manufacturer’s instruction, and analyzed by electrophoresis on a Bolt 4%–12% Bis-Tris Plus gel.

### 4.8. Other Methods

Quantitation of MDA-HDL: MDA-HDL was quantitated by using an OxiSelect Human Oxidized HDL ELISA Kit (Cell Biolabs, Inc., San Diego, CA, USA). For this assay, the HDL fraction was first isolated from the plasma by the dextran sulfate-Mg^2+^ precipitation method. It was then diluted 1:50 with Assay Diluent, and an aliquot (100 µL) of the diluted HDL was used for the determination of MDA-HDL.

Purification of LDL/VLDL: One ml of plasma from a healthy male subject was used for the LDL/VLDL purification. The LDL/VLDL was purified by using an LDL/VLDL Purification Kit (Cell Biolabs, Inc.), and its activity was determined by performing the Fe-ROMs test after dialysis against a phosphate-buffered saline (pH 7.4).

### 4.9. Statistical Analysis

Data were presented as mean ± standard deviation (SD) and subjected to a one-way analysis of variance ANOVA with Tukey HSD post-hoc comparisons. The Pearson’s correlation coefficient was used to identify the association between the levels obtained by the d-ROMs and Fe-ROMs tests. It was also conducted for the association between the levels quantified by the Fe-ROMs test and the MDA-HDL ELISA kit after the HDL fractionation. The analysis was performed using SPSS software version 24 (IBM Japan, Tokyo, Japan), and the statistical level of significance was set at 0.05.

## 5. Conclusions

The Fe-ROMs test was demonstrated to be able to assess oxidative stress with ease by using just a small amount of human plasma. Further, oxidized HDL can be estimated from Fe-ROMs levels in plasma. Emerging evidence shows that the anti-atherogenic role of HDL is not simply defined by the plasma HDL-C level but rather by a functional property of HDL. Because the functionality of HDL is affected by the oxidization of HDL components, the measurement of oxHDL must be useful for the assessment of the risk of cardiovascular disease.

## Figures and Tables

**Table 1:** Cholesterol concentrations of the major classes and subclasses of lipoproteins in plasma and HDL fractions.

Class	Sub-Class	Cholesterol (mg/dL)	
		Plasma	HDL
CM (>80 nm)		1.73	0.01
VLDL (30–80 nm)	Large	24.81	0.03
	Medium	8.21	0.01
	Small	7.31	0.01
LDL (16–30 nm)	Large	34.02	0.03
	Medium	57.74	0.02
	Small	21.12	0.06
	Very small	7.73	0.23
HDL (8–16 nm)	Very large	3.66	2.36
	Large	22.76	20.63
	Medium	22.48	20.32
	Small	13.69	12.17
	Very small	5.11	4.14
Total		230.36	60.62

HDL was isolated from the plasma of a healthy subject by the use of the dextran sulfate-Mg^2+^ precipitation method. HDL, high-density lipoprotein; LDL, low-density lipoprotein; VLDL, very low-density lipoprotein.

**Table 2:** The cholesterol concentrations of the major classes of lipoproteins in four plasma samples containing low or high levels of Fe-ROMs.

Subject	Fe-ROMs Level 505 nm (mOD/min)	Cholesterol (mg/dL)				
		Total	CM	VLDL	LDL	HDL
			>80 nm	30–80 nm	16–30 nm	8–16 nm
1	Low (19.5)	46.36	0.02	0.06	0.29	45.99
2	Low (19.8)	47.87	0.01	0.05	0.30	47.50
3	High (31.8)	46.00	0.01	0.05	0.33	45.61
4	High (28.0)	41.49	0.01	0.07	0.31	41.11

**Table 3:** The cholesterol concentrations of the subclasses of HDL in four plasma samples containing low or high levels of Fe-ROMs.

Subject	HDL Cholesterol (mg/dL)						
	Very Large		Large	Medium	Small	Very Small	
	Fraction 14	Fraction 15	Fraction 16	Fraction 17	Fraction 18	Fraction 19	Fraction 20
1	0.07	1.25	9.83	18.02	12.08	3.68	1.08
2	0.02	1.65	11.57	17.90	11.58	3.67	1.11
3	0.05	1.66	10.39	16.85	11.80	3.70	1.15
4	0.03	2.33	10.89	14.07	9.57	3.16	1.06

## Supplementary Materials

Supplementary materials can be found at www.mdpi.com/1422-0067/18/2/454/s1.

## Abbreviations

DHP	Dihydropyridine
d-ROMs	Diacron reactive oxygen metabolites
ELISA	Enzyme-linked immunosorbent assay
HDL	High-density lipoprotein
HPLC	High-performance liquid chromatography
LDL	Low-density lipoprotein
MDA	Malondialdehyde
oxLDL	Oxidatively modified low-density lipoprotein
oxHDL	Oxidized high-density lipoprotein
PUFA	Polyunsaturated fatty acid
ROS	Reactive oxygen species
SDS PAGE	Sodium dodecyl sulfate poly-acrylamide gel electrophoresis
VLDL	Very low-density lipoprotein
