# Supplementary material for: The Application of a Modified d-ROMs Test for Measurement of Oxidative Stress and Oxidized High-Density Lipoprotein
Source: Int J Mol Sci. 2017 Feb 21;18(2):454. doi: 10.3390/ijms18020454 (PMC5343988; doi:10.3390/ijms18020454)
Supplement: Supplementary file 1 [file ijms-18-00454-s001.pdf]

Supplementary Materials: The Application of a Modified d-ROMs Test for Measurement of Oxidative Stress and Oxidized High-Density Lipoprotein

Fumiaki Ito, Tomoyuki Ito, Chinatsu Suzuki, Tomoyo Yahata, Kazuyuki Ikeda and Kenji Hamaoka

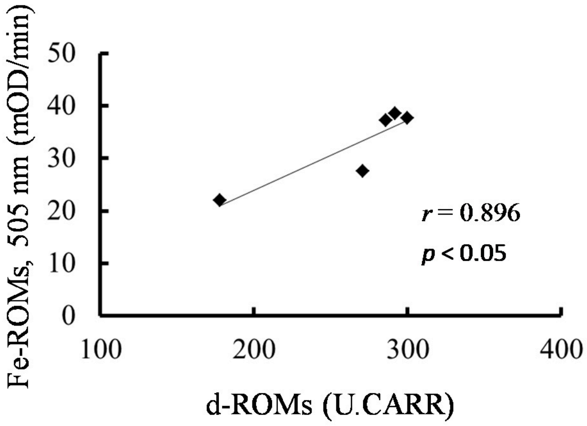

Figure S1. The correlation of Fe-ROMs values with diacron-reactive oxygen metabolites (d-ROMs) values. The oxidative stress was evaluated by measuring plasma samples from five healthy subjects. The rates of increase in absorbance at 505 nm were determined in the presence (Fe-ROMs test) and absence (d-ROMs test) of Fe^{2+}, as described in the Materials and Methods. The correlation between the values obtained from the d-ROMs and Fe-ROMs tests was expressed by Pearson's correlation coefficient ($r = 0.896$, $p < 0.05$). U.CARR, Carratelli units.

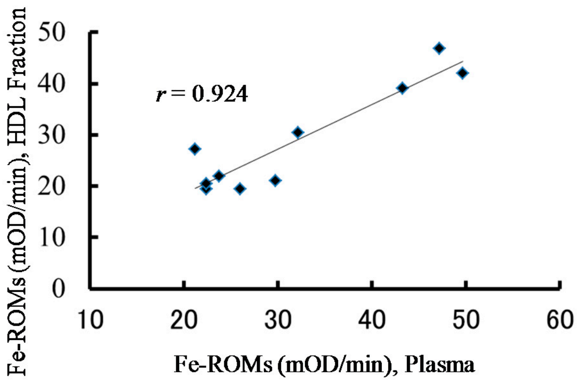

Figure S2. The correlation between the Fe-ROMs levels in the plasma and HDL fraction. HDL fractions were isolated from the plasma of 10 male patients with acute febrile disease, as described in Figure 4. The Fe-ROMs values were then measured by using these plasma samples and isolated HDL fractions. The correlation between the Fe-ROMs levels in the plasma and isolated HDL fractions is expressed by Pearson's correlation coefficient ($r = 0.924$, $p < 0.001$).

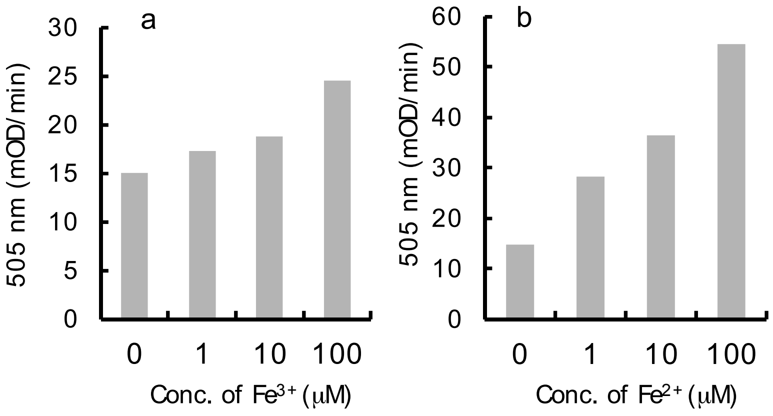

Figure S3. The dose-dependent effect of Fe^{3+} (**a**); and Fe^{2+} (**b**) on the measurement of reactive oxygen metabolites. The rates of increase in absorbance at 505 nm after the addition of plasma and *N*,*N*′-diethyl-*p*-phenylenediamine were determined in the presence of the indicated concentrations of iron ions. The plasma sample was obtained from a healthy subject (the concentration of iron ions in the plasma is 0.45 mM). A similar dose-dependent effect was obtained by using the plasma sample from another healthy subject (the concentration of iron ions in the plasma is 0.28 mM).
